# Inhibition of Shear-Induced Platelet Aggregation by Xueshuantong via Targeting Piezo1 Channel-Mediated Ca^2+^ Signaling Pathway

**DOI:** 10.3389/fphar.2021.606245

**Published:** 2021-03-22

**Authors:** Lei Liu, Qiongling Zhang, Shunli Xiao, Zhengxiao Sun, Shilan Ding, Ying Chen, Lan Wang, Xiaojie Yin, Fulong Liao, Lin-Hua Jiang, Mei Xue, Yun You

**Affiliations:** ^1^ Institute of Chinese Materia Medica, China Academy of Chinese Medical Sciences, Beijing, China; ^2^ Sino-UK Joint Laboratory of Brain Function and Injury, Xinxiang Medical University, Xinxiang, China; ^3^ School of Biomedical Sciences, Faculty of Biological Sciences, University of Leeds, Leeds, United Kingdom; ^4^ XiYuan Hosipital, China Academy of Chinese Medical Sciences, Beijing, China

**Keywords:** xueshuantong, shear, platelet aggregation, piezo1 channel, Ca^2+^

## Abstract

XueShuanTong (XST) comprising therapeutically active ginsenosides, a lyophilized extract of *Panax notoginseng* roots, is extensively used in traditional Chinese medicine to treat ischemic heart and cerebrovascular diseases. Our recent study shows that treatment with XST inhibits shear-induced thrombosis formation but the underlying mechanism remained unclear. This study aimed to investigate the hypothesis that XST inhibited shear-induced platelet aggregation via targeting the mechanosensitive Ca^2+^-permeable Piezo1 channel by performing platelet aggregation assay, Ca^2+^ imaging and Western blotting analysis. Exposure to shear at physiologically (1,000–2000 s^−1^) and pathologically related rates (4,000–6,000 s^−1^) induced platelet aggregation that was inhibited by treatment with GsMTx-4. Exposure to shear evoked robust Ca^2+^ responses in platelets that were inhibited by treatment with GsMTx-4 and conversely enhanced by treatment with Yoda1. Treatment with XST at a clinically relevant concentration (0.15 g L^−1^) potently inhibited shear-induced Ca^2+^ responses and platelet aggregation, without altering vWF-mediated platelet adhesion and rolling. Exposure to shear, while resulting in no effect on the calpain-2 expression in platelets, induced calpain-2-mediated cleavage of talin1 protein, which is known to be critical for platelet activation. Shear-induced activation of calpain-2 and cleavage of talin1 were attenuated by treatment with XST. Taken together, our results suggest that XST inhibits shear-induced platelet aggregation via targeting the Piezo1 channel to prevent Piezo1-mediated Ca^2+^ signaling and downstream calpain-2 and talin1 signal pathway, thus providing novel insights into the mechanism of the therapeutic action of XST on platelet aggregation and thrombosis formation.

## Introduction

Cardiovascular diseases (CVD) have emerged as one of the most major causes of mortality and morbidity worldwide. It is widely accepted that platelets play a pivotal role in hemostasis and blood clotting at the sites of vascular injuries and, furthermore, platelets represent a significant linkage between thrombosis, inflammation and atherogenesis (Papapanagiotou et al., 2016). Hemostasis maintains the regulation of vascular integrity and blood flow and, if overwhelmed, can lead to clot formation and blood vessel occlusion. In addition to platelets, endothelial cells and circulating coagulation proteins, blood flow is a crucial factor in vascular hemostasis and thrombosis (Holinstat, 2017; Koupenova et al., 2017). It is known that platelet adhesion to immobilized von Willebrand factor (vWF) and subsequent formation of platelet-derived micro-particles are mediated by glycoprotein Ibα (GPIbα) under high shear stress (Kroll, 2010). Flow-induced shear stress has significant influence on arterial thrombus formation. In particular, increasing concentration of platelets and/or activation of platelets at the vessel wall enhance growth and stability of thrombi, which may result in a fatal narrowing of the arterial lumen. The efficacy of several antithrombotic agents is shear stress-dependent (Maxwell et al., 2007; Sakariassen et al., 2015). Arterial thrombosis is the major cause of myocardial infarction and stroke, while venous thrombosis leads to venous thromboembolism and pulmonary embolism. Structurally, arterial and venous thrombi are distinct. Arterial thrombi are rich in platelets and form at the sides of, or around the ruptured atherosclerotic plaques, and venous thrombi are rich in fibrin and red blood cells (Koupenova et al., 2017). Previous studies reported that shear-induced adhesion of platelets at exposed sub-endothelial collagen/vWF allows platelets to aggregate (Maxwell et al., 2007; Cosemans et al., 2013). A recent study has reported that the PI3K-C2α lipid kinase mediates shear-dependent regulation of platelet adhesive function via altering the internal membrane structures (Mountford et al., 2015). Piezo1 was first showed to form a mechanosensitive or mechanically activated Ca^2+^-permeable cation channel in mouse neuroblastomas cell (Coste et al., 2010). The Piezo1 channel opens by shear-induced bilayer tension (Lewis and Grandl, 2015; Cox et al., 2016; Syeda et al., 2016) and changes in membrane curvature (Guo and MacKinnon, 2017; Lin et al., 2019) and mediates Ca^2+^ influx and activation of downstream signal pathways (Li et al., 2014; Ranade et al., 2014). Numerous studies have now confirmed that the Piezo1 channel senses diverse mechanical stimuli and regulates a wide range of cell functions and biological processes (Li et al., 2014; Cahalan et al., 2015; Hyman et al., 2017; Wu et al., 2017; Romac et al., 2018; Zhong et al., 2018). A recent study suggests that the Piezo1 channel plays a role in mediating Ca^2+^ entry into platelets and thrombus formation under arterial shear stress (Ilkan et al., 2017).

Currently available antithrombotic drugs, including the widely used antiplatelet agents and anticoagulants, are associated with significant bleeding risk. There is growing awareness of the use of herbs in the prevention of platelet aggregation and treatment of related CVDs. *Panax notoginseng* (Burk.) F. H. Chen, an important herb used in traditional Chinese medicine (TCM), has been reported to have beneficial effects, including promotion of blood circulation, removal of blood stasis, relief of pain and hemostasis. It has been extensively used since it was discovered in the Ming dynasty and its medical functions were described in the Compendium of Materia Medica (Ben Cao Gang Mu). The most important and active ingredients of panax notoginseng are saponins in a variety of monomeric species, including ginsenosides (Rg_1_, Rb_1_, Re, Rd, Rb_2_, Rb_3_, Rc) and notoginsenosides (R_1_, R_2_, R_3_ and R_6_). Studies have shown that *Panax notoginseng saponins* (PNS) have the effects of anti-myocardial infarction (Liu et al., 2014), antithrombotic (Lau et al., 2009; Liu et al., 2018), anti-inflammatory (Chang et al., 2007), antioxidant (Peiran et al., 2017) and amelioration of microcirculation disorders (Han et al., 2017). An early study reported that PNS had an inhibitory effect on shear-induced platelet aggregation (Liao, 1997), but the underlying mechanism of action remains unclear. Xueshuantong used in TCM for the prevention and treatment of thrombosis contain high content of PNS. Our recent studies show that XST inhibited platelet activation and adhesion to injured endothelial cells (ECs) *in vitro* (Han et al., 2019) and also inhibited tail thrombosis formation induced by κ-carrageenan in rats and ameliorated vascular hemodynamic status (Liu et al., 2020). Interestingly, such an inhibitory effect of XST was shear-dependent but the underlying mechanism remained unclear. In the present study, we provide evidence to support that XST inhibits shear-induced platelet aggregation via the mechanosensitive Piezo1 channel.

## Materials and Methods

### Reagents

XueShuanTong, with the Chinese NMPA drug ratification number of GuoYaoZhunZi-Z20025652, has been manufactured as a sterile and nonpyrogenic lyophilized solid for intravenous administration. Each gram of XST was prepared from 15.4 g of Sanqi (*P. notoginseng* (Burk.) F. H. Chen). XST used in this study was obtained from Guangxi Wuzhou Pharmaceutical Group with lot No.17081507 (Wuzhou, Guangxi Zhuang Autonomous Region, China).

Yoda1 (448947-81-7) and GsMTx-4 peptide (STG-100) were purchased from Tocris Bioscience (Bristol, United Kingdom) and Alomone Labs (Jerusalem, Israel), respectively. XST was dissolved in saline at 30 mg mL^−1^ storage concentration; Yoda1 in DMSO at 10 mM storage concentration; GsMTx-4 in water at 100 μM storage concentration. Storage concentration of XST, GsMTx-4 and Yoda1 was diluted by saline when tested. Yoda1 was diluted to the final concentration of 25 μM (0.25% v/v DMSO). Previous studies revealed that 0.25% (v/v) DMSO showed neither obvious influences on ADP, collagen or thrombin induced platelets aggregation (Yi et al., 2017; Wang et al., 2019) nor shear-induced platelet aggregation (Supplementary Figure S1).

Calcein-AM and Fluo3-AM were from Invitrogen (Carlsbad, CA, United States) and Dojindo Laboratories (Kumamoto, Japan), respectively. Type I collagen (#385) was from Chron-Log Corp (Havertown, PA, United States), and von Willebrand Factor (Z0409) was from Hematologic Technologies (Essex Junction, VT, United States). Anti-Piezo1 antibody (APC-087) was from Alomone Labs, and anti-calpain2 antibody (Cat No. 11472-1-AP), anti-talin1 antibody (Cat No. 14168-1-AP), anti-vinculin antibody (Cat No. 66305-1-Ig), anti-β-actin (Cat No. 66009-1-Ig), horseradish peroxidase (HRP)-conjugated Affinipure goat anti-mouse IgG antibody (SA00001-1) and HRP-conjugated Affinipure goat anti-rabbit IgG antibody (SA00001-2) were from ProteinTech (Rosemont, IL, United States).

### Platelet Preparation

The procedures of blood withdrawal from male Sprague-Dawley rats (250 ∼ 270 g) were performed in accordance with the guidelines approved by the Animal Care and Use Committee of Institute of Chinese Materia Medica, China Academy of Chinese Medical Sciences (Beijing, China). Rats were obtained from the Experimental Animal Center, Charles River Laboratories (Beijing, China) [SCXK2016-0006], and housed at 15–28°C and humidity of 45–55%, with free access to food and drink, for 3 days before used. Rats were anesthetized by injection of 60 mg kg^−1^ sodium pentobarbital at a concentration of 3%, and 8 ml of whole blood samples were withdrawn from the abdominal aorta per rat and anticoagulated using 109 mM sodium citrate (1:9). For platelet aggregation assay, platelet-rich plasma (PRP) was obtained by centrifugation of the blood sample at 200 × *g* for 10 min. For Ca^2+^ imaging and Western blotting, platelets were prepared as follows: PRP was mixed with ACD (2.5% trisodium citrate, 2.0% glucose, and 1.5% citric acid) and EDTA (100 mM) in a ratio of 9:1:0.2, and centrifuged at 400 × *g* for 10 min. Platelets were suspended with Tyrode’s solution (138 mM NaCl, 3.3 mM NaH_2_PO_4_, 2.9 mM KCl, 1 mM MgCl_2_, 5.5 mM glucose, and 20 mM HEPES, pH 7.2) and were centrifuged again at 400 × *g* for 10 min. Platelets were re-suspended in Tyrode’s solution. Preparation of platelets was conducted at room temperature.

### Platelet Aggregation Assay

The High Shear BioFlux 48-well plates (Fluxion Biosciences, Alameda, CA, United States) were used. The microfluidic channels were coated with 40 μg m L^−1^ collagen for 1 h, and then blocked with 0.5% bovine serum albumin (BSA) in phosphate-buffered saline (PBS) at room temperature. The platelet density in PRP was adjusted to 5.0 × 10^10^/L with Tyrode’s solution, and the platelet suspension was incubated with Calcein-AM (5 µM) for 1 h at room temperature in dark. Calcein-AM-labelled platelets were treated with XST (0.15 g L^−1^ or 0.6 g L^−1^), GsMTx-4 (2.5 µM), Yoda1 (25 µM), Yoda1 (25 µM) plus XST (0.15 g L^−1^ or 0.6 g L^−1^), or vehicle (saline) for 10 min at 37°C in dark.

Platelets were perfused to the microfluidic channels with incremental pressures of 0.13, 0.33, 0.66, 1.32, 2.64 psi for 5 min, which resulted in wall shear rates of 400, 1,000, 2,000, 4,000, and 8,000 s^−1^, respectively, and the whole shear flow continued for 25 min. Fluorescent micrographs of adhesion and aggregation of platelets were captured by a time-lapse inverted microscope (wavelength FITC module; exposure time: 400 ms) using an Axio Observer 7 (Carl Zeiss AG, Oberkochen, Germany) and a C11440-42U30 digital camera (Hamamatsu Photonics, Shizuoka, Japan). The BioFlux-1000Z system (Fluxion Biosciences, Alameda, CA, United States; Conant et al., 2011) was utilized to control the shear rate and image acquisition settings. Platelet aggregation rates were determined by the fluorescence intensity using bio flux Montage software (Fluxion Biosciences, Alameda, CA, United States). Three independent experiments were carried out with at least triplicate.

### Platelet Adhesion and Rolling Assays

The High Shear BioFlux 48-well plates were utilized. The microfluidic channels were coated with 70 μg m L^−1^ vWF for 1 h and then blocked with 0.5% (v/v) BSA in PBS for 10 min at room temperature. The sodium citrate anti-coagulated whole blood was treated with XST (0.15 g L^−1^ or 0.6 g L^−1^) or vehicle (saline) for 10 min at 37°C prior to perfusion at a shear rate of 750 s^−1^ (shear stress of 3 Pa) for 10 min. Subsequently, the channels were washed for 5 min at a shear rate of 1,000 s^−1^ with HBSS (with calcium and magnesium) until all red and white blood cells were clear of the channels. Only platelets were loaded on the surface of channels coating with vWF protein. The rolling of platelets under a shear rate of 6,000 s^−1^ with HBSS containing saline or XST (0.15 g L^−1^ or 0.6 g L^−1^) was recorded by time-lapse imaging every second for 30 s. The platelet adhesion and rolling speed were measured using bio flux Montage software (Fluxion Biosciences, Alameda, CA, United states). Measurements of distance/time were taken for 10 rolling platelets in each field. Three independent experiments were carried out same as described above.

### Ca^2+^ Imaging in Platelets

The effects of XST on shear-induced Ca^2+^ influx were assessed using bio flux 24-well plates with a two-inlet well (A and B) to one-outlet well (O) per channel design (Supplementary Figure S2). The 24-well microfluidic channels were coated with 40 μg mL^−1^ collagen for 1 h and then blocked with 0.5% BSA in PBS for 10 min at room temperature. Platelets were adjusted to a density of 3 × 10^8^/ml with Tyrode’s solution containing 1 mM CaCl_2_ and incubated with 5 µM Fluo3-AM for 45 min at 37°C in dark. All inlet A wells were filled with Fluo3-AM-labeled platelets and inlet B wells were added with 1 ml of XST (0.15 g L^−1^), GsMTx-4 (2.5 µM), Yoda1 (25 µM), or Yoda1 (25 µM) together with XST or saline. Flow was initiated from the inlet A wells at a shear rate of 200 s^−1^ (shear stress of 0.2 Pa) for 2 min to load platelets on the surface of channels, accompanied with sequential perfusion from the inlet B wells starting automatically and continuing for 10 min at a shear rate of 1,000 s^−1^ (shear stress of 1 Pa). Fluorescent images were captured every 30 s for 12 min. Fluorescent intensity was determined by bio flux Montage software (Fluxion Biosciences, Alameda, CA, United States). The fluorescent intensity (F) was corrected for background before normalized to the initial fluorescence intensity (F_0_) to yield the F/F_0_ values. Three independent experiments were carried out same as described above.

### Western Blotting

For shear conditions, platelets were incubated with saline, XST (0.15 g L^−1^ or 0.6 g L^−1^), GsMTx-4 (2.5 µM), Yoda1 (25 µM), or Yoda1 (25 µM) plus XST for 10 min before added into BioFlux 48-well plates and subjected to shear at a rate of 4,000 s^−1^ for 30 min. For static conditions, platelets were incubated with same volume of saline and subjected to no flow for 30 min. Platelets were collected by centrifugation for 10 min at 1,200 × *g* and lyzed in radio-immune precipitation assay buffer containing 1 mM phenylmethylsulfonyl fluoride. After centrifugation at 13,000 × *g* and 4°C for 5 min, the protein concentrations in the supernatants were measured using a bicinchoninic acid assay kit (Thermo Fisher Scientific, MA, United States) according to the manufacturer’s instructions. Proteins of 30–40 µg were separated by electrophoresis on 10% sodium dodecyl sulfate-polyacrylamide gels, and subsequently transferred onto polyvinylidene difluoride membranes. Membranes were blocked with 5% skim milk in Tris-buffered saline containing Tween 20 (TBST) (20 mM Tris-HCl, pH 7.5, 137 mM, NaCl, and 0.1% Tween 20) at room temperature for 2 h, and incubated with primary anti-Piezo1 (1:600), anti-Calpain-2 (1:2,000), anti-talin1 (1:500), anti-vinculin (1:5,000), anti-β-actin (1:5,000), or anti-GAPDH antibody (1:5,000) at 4°C overnight. After washing with TBST, the membranes were incubated with HRP-conjugated secondary antibodies for 2 h at room temperature. Proteins were visualized by enhanced chemiluminescence (ECL) (Millipore, MA, United States) and imaged using an ECL detection system (Syngene, Cambridge, United Kingdom). The protein density was analyzed by ImageJ software. Three independent experiments were carried out same as described above.

### Statistical Analysis

All the platelet aggregation rates data were expressed as mean ± SD, and two-way analysis of variance (ANOVA) followed by post-hoc Tukey’ test was performed; the other data were expressed as mean ± SEM, and one-way ANOVA followed by post-hoc Tukey’ test was performed using SPSS 20.0 software (IBM, Armonk, NY, United States). At least three independent experiments were carried out. *p* < 0.05 was considered statistically significant.

## Results

### Chemical Quality Control of XueShuanTong

**FIGURE 1:**
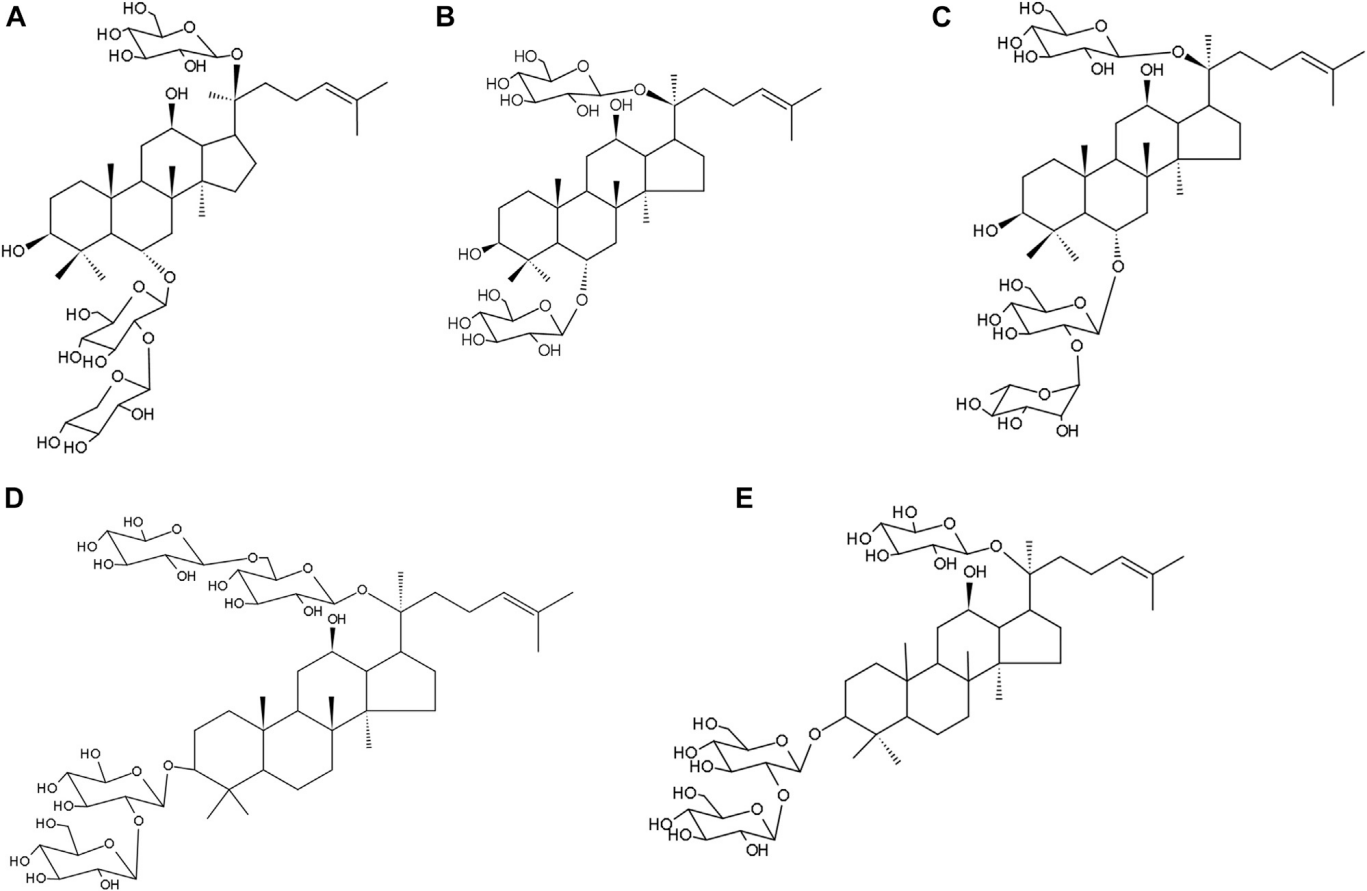
The five major ginsenosides in XueShuanTong and their chemical structures. **(A)** Notoginsenoside R_1_ (12.6%). **(B)** Ginsenoside Rg_1_ (43.5%). **(C)** Ginsenoside R_e_ (5.6%). **(D)** ginsenoside Rb_1_ (27.8%). **(E)** ginsenoside R_d_ (2.3%).

Each gram of XST was prepared from 15.4 g of Sanqi (Panax notoginseng (Burk.) F. H. Chen). XST used in this study was obtained from Guangxi Wuzhou Pharmaceutical Group with lot No. 17081507 (Wuzhou, Guangxi Zhuang Autonomous Region, China). XST contain five major saponins ginsenoside Rg_1_ (43.5%), ginsenoside Rb_1_ (27.8%), notoginsenoside R_1_ (12.6%), ginsenoside Re (5.6%) and ginsenoside R_d_ (2.3%), and these five major ginsenosides make up 91.8%. The chemical structures of these five ginsenosides in XST are shown in Figure 1 and the chemical analysis data of XST is provide by the company shown in Supplementary Figure S3. An offline 2D LC/QTOF-Fast DDA approach was developed with the view of systematic exposure and characterization of the minor saponins contained in XST. As many as 143 minor saponins were separated and characterized (Yang et al., 2016), the content of trace components is almost clear till now and make up range of 9 ∼ 12% in XST in the quality control report.

### Shear Induces Platelet Aggregation via Activating the Piezo1 Channel

**FIGURE 2:**
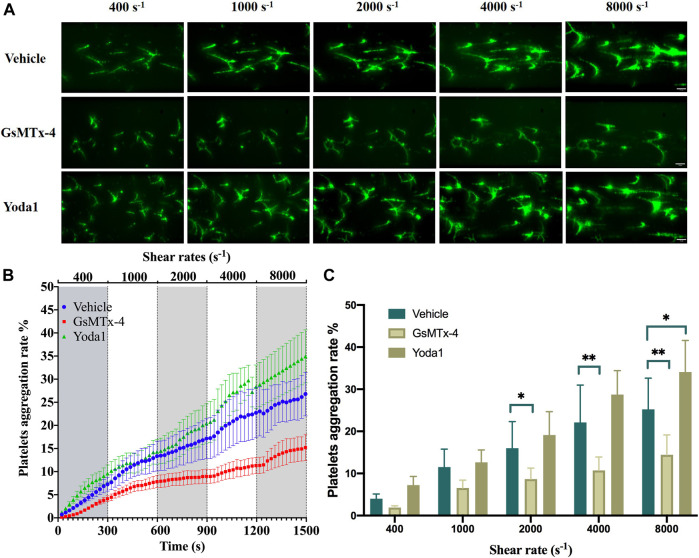
Role of the Piezo1 channel in shear-induced platelet aggregation. **(A)** Representative images showing effects of treatment with GsMTx-4 (2.5 µM) and Yoda1 (25 µM) on platelet aggregation under shear rates of 400, 1,000, 2,000, 4,000 and 8,000 s^−1^. The scale bar is 50 µm. **(B)** Mean time-course of plate aggregation from three independent experiments under the conditions shown in **(A)**. **(C)** Mean platelet aggregation rate at the end of exposure to indicated shear rates. *p < 0.05 and **p < 0.01, analyzed by two-way ANOVA and post-hoc Tukey’s test.

A recent study has reported the expression of the Piezo1 channel in platelets as a mechanosensitive ion channel that can be activated by shear (Ilkan et al., 2017). We were interested in whether shear enhanced platelet aggregation and, if it did, whether such an effect occurred via activating the Piezo1 channel. Subendothelial collagen exposure is a thrombogenic factor inducing platelet aggregation *in vivo*. We assessed the effect of shear on collagen-induced platelet aggregation by applying shear to platelets on collagen-coated surface, starting at a shear rate of 400 s^−1^ and increasing to 1,000 and 2,000 s^−1^ observed under physiological conditions, and further to 4,000 and 8,000 s^−1^ under pathological conditions. As shown in Figures 2A,B, exposure to shear resulted in a significant and shear rate dependent increase in platelet aggregation. There were interactions between shear rate and treatment with GsMTx-4 or Yoda1 on platelet aggregation (F = 2.343, *p* = 0.029) by two-way ANOVA, which implied that GsMTx-4 or Yoda1 influenced shear induced platelet aggregation. Treatment of platelets with 2.5 µM GsMTx-4, a peptide known to inhibit the Piezo1 channel (Bae et al., 2011; Hara et al., 2012; Pathak et al., 2014), attenuated shear induced platelet aggregation at shear rate of ≥2000 s^−1^ (Figure 2C), and compared with vehicle, GsMTx-4 reduced platelet aggregation significantly (*p* < 0.05 or *p* < 0.01). Conversely, treatment with 25 µM Yoda1, a Piezo1 channel agonist, enhanced shear-induced platelet aggregation at shear rate of 8,000 s^−1^(*p* = 0.018), though such a stimulatory effect was not statistically significant at shear rate ranged from 400 to 4,000 s^−1^ (Figures 2B,C).

### XueShuanTong Inhibits Shear-Induced Platelets Aggregation

**FIGURE 3:**
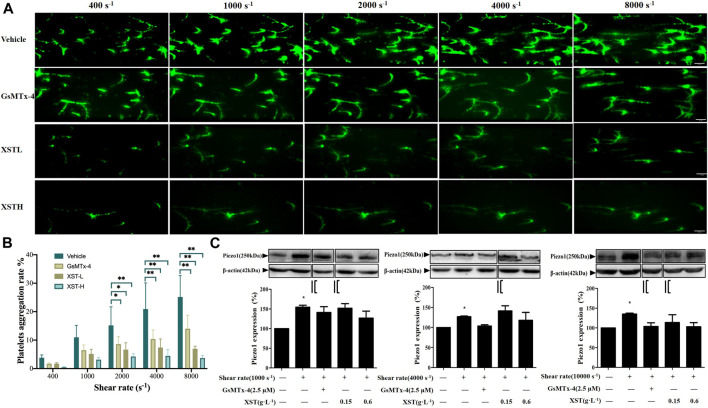
Effects of treatment with XST on shear-induced platelet aggregation. **(A)** Representative images showing effects of treatment with XST at 0.15 g L^−1^ (XSTL) or 0.6 g L^−1^ (XSTH) and GsMTx-4 (2.5 µM) on platelet aggregation. The scale bar 50 µm. **(B)** Mean platelet aggregation rate at the end of exposure to indicated shear rates under the conditions shown in **(A)**. *p < 0.05 and **p < 0.01, analyzed by two-way ANOVA and post-hoc Tukey’s test. **(C)** Western blotting analysis of the Piezo1 expression in platelets under static conditions or after exposure to indicated shear alone or together with GsMTx-4 or XST, and the grouping and splicing was highlighted by black lines. Top: representative blots; bottom: mean data from three independent experiments by one-way ANOVA. *p < 0.05 compared to platelets under static conditions.

We next investigated whether treatment with XST affected shear-induced platelet aggregation. There were interactions between shear rate and treatment with XST or GsMTx-4 on platelet aggregation by two-way ANOVA (F = 2.306, *p* = 0.024), which indicated that XST or GsMTx-4 affected shear induced platelet aggregation. Treatment with XST at a concentration of 0.15 g L^−1^ (XST-L), prior to and during exposure to shear, strongly reduced platelet aggregation induced by shear at a rate exceeding to 2000 s^−1^. Treatment with XST at a relatively high concentration of 0.6 g L^−1^ (XST-H) resulted in similar inhibition of shear-induced platelet aggregation (Figure 3A; Supplementary Videos S1A–D). Figure 3B shows the mean platelet aggregations rate at the end of exposure to shear with the rates of 400, 1,000, 2,000, 4,000 and 8,000 s^−1^ under indicated conditions (vehicle, GsMTx-4, XST-L and XST-H). Under the shear rate level higher than 2,000 s^−1^, multiple comparison by post-hoc Tukey’ test showed that compared to the vehicle group, treatment with GsMTx-4, XST-L or XST-H significantly inhibited platelet aggregation induced by physiological 2,000 s^−1^ or pathophysiological shear (4,000–8,000 s^−1^) (Figure 3B) (*p* < 0.05 or *p* < 0.01).

**FIGURE 4:**
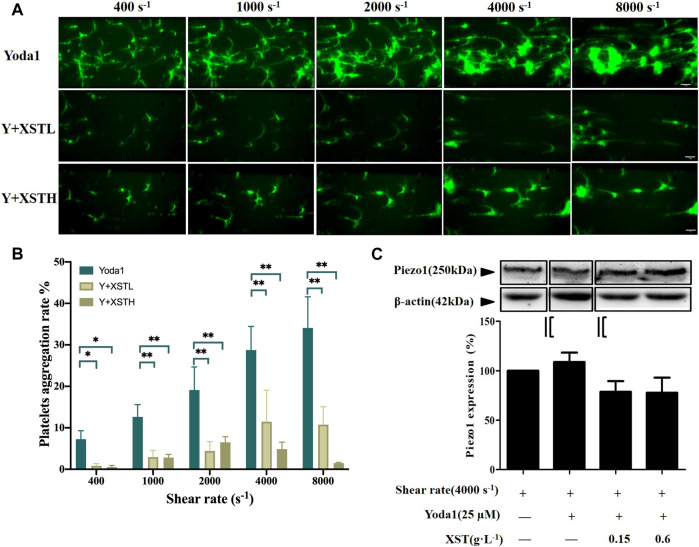
Effects of XST on platelet aggregation induced by shear stress and Yoda1. **(A)** Representative images showing the effects of treatment with Yoda1 (25 µM) alone or together with XST at 0.15 g L^−1^ (XSTL) or 0.6 g L^−1^ (XSTH) on shear-induced platelet aggregation. The scale bar 50 µm. **(B)** Mean platelet aggregation rate at the end of exposure to indicated shear rates. ^#^ *p* < 0.05 and ^##^ *p* < 0.01, analyzed by two-way ANOVA and post-hoc Tukey’s test. **(C)** Western blotting analysis of the Piezo1 expression in platelets under shear rate of 4,000 s^−1^ and exposure to Yoda1 (25 μM) alone or Yoda one together with XST, and the grouping was highlighted by black lines. Although shear induced platelets aggregation was enhanced by Yoda1, however, exposure to Yoda1 alone, or Yoda1 together with XST resulted in no significant effect on the Piezo1 expression. Top: representative blots; bottom: mean data from three independent experiments.

We performed Western blotting to examine the Piezo1 protein level in platelets under static and shear conditions and also platelets treated with GsMTx-4, XST-L and XST-H under shear. Compared with static conditions, the Piezo1 protein level was significantly enhanced after exposure to shear (1,000, 4,000 or 10,000 s^−1^), which may contribute to shear-induced platelet aggregation. Treatment with GsMTx-4, XST-L or XST-H showed no effects on the Piezo1 protein expression under high shear rate (Figure 3C), suggesting that XST inhibits shear-induced platelet aggregation mainly via preventing activation of the Piezo1 channel. In order to interpret the piezo1 channel function in shear-induced platelet aggregation step by step, we grouped the Western blotting data in both Figures 3C, 4C which were highlighted by black lines and the original images are supplemented. Aspirin has neither effects on shear-induced platelets aggregation nor on piezo1 expression, and the results of aspirin were shown as the Supplementary Figure S4.

We also examined the effects of treatment with XST-L or XST-H on shear-induced platelet aggregation in the presence of Yoda1, as shown in Figure 4A and Supplementary Videos S1E–G. Figure 4B shows the mean platelet aggregations rate at the end of exposure to indicated shear rates under these conditions. There were very significant interactions between shear stress and treatment with Yoda1 or Yoda1 together with XST on platelet aggregation by two-way ANOVA (F = 10.32, *p* < 0.001), which suggested that Yoda1 together with XST affected shear induced platelet aggregation. At shear rate of 400, 1,000, 2,000, 4,000 and 8,000 s^−1^, multiple comparison by post-hoc Tukey’ test shows that treatment with XST-L and XST-H significantly inhibited platelet aggregation induced by shear and Yoda1 (*p* < 0.05 or 0.01). We also examined using Western blotting the Piezo1 protein level in platelets exposed to shear at a rate of 4000 s^−1^, in the presence of Yoda1 or Yoda1 together with XST. Exposure to Yoda1 alone, or Yoda1 together with XST resulted in no significant effect on the Piezo1 expression (Figure 4C). These results provide further evidence to support that XST inhibits shear-induced platelet aggregation mainly via the Piezo1 channel function.

Interactions between platelets and vWF play an important role in shear-induced platelet aggregation under high shear rates (>5,000 s^−1^) and, in addition, the arrest of flowing platelets (adhesion and rolling) on vWF surface are thought to be mediated by the interactions between the A1 domain of vWF and the platelet receptor GPIbα (Ruggeri et al., 2006). We were interested in whether XST can affect shear-induced platelet aggregation through altering the interactions of platelets with vWF. To address, platelet adhesion and rolling on vWF-coated surface under high shear rate of 6,000 s^−1^ was assessed. Platelets adhered to vWF-coated surface as small aggregates, which were different from thrombi formed on collagen-coated surface. Neither platelet adhesion nor rolling was significantly altered by treatment with XST (Supplementary Figure S5). Taken together, our results suggest that XST preferentially inhibits shear-induced platelet aggregation with minimal effect on vWF mediated platelet adhesion and rolling.

### XueShuanTong Inhibits Shear-Induced Piezo1-Mediated Ca^2+^ Signaling in Platelets

**FIGURE 5:**
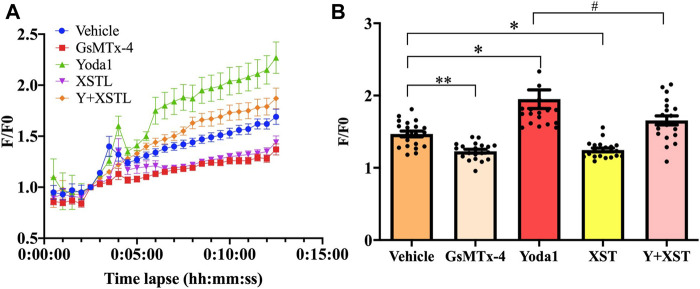
Effects of treatment with XST on shear induced Ca^2+^ responses. **(A)** Time-course of changes in intracellular Ca^2+^ mean levels (F/F_0_) in platelets subjected to a shear rate of 1,000 s^−1^ without (vehicle) and with treatment with GsMTx-4 (2.5 µM), Yoda1 (25 µM), XST (0.15 g L^−1^), and XST together with Yoda1. **(B)** Effects of treatment with GsMTx-4, Yoda1, XST or XST together with Yoda1 on shear-induced Ca^2+^ responses in platelets at time point of 8.5 min. Three independent experiments were undertaken. *p < 0.05 and **p < 0.01 compared to the vehicle group; ^#^ *p* < 0.05 compared to platelets treated with Yoda1 alone, analyzed by one-way ANOVA and post-hoc Tukey’s test.

To provide direct evidence to support the notion that XST inhibits shear-induced platelet aggregation via targeting the Piezo1 channel, we next examined the effect of treatment with XST on shear-induced Ca^2+^ responses in platelets, using Ca^2+^ fluorescent indicator Fluo3-AM. The single platelet fluorescent intensity (F) was corrected for background before normalized to the initial fluorescence intensity (F_0_) to yield the F/F_0_ values, three independent experiments were carried with totally 20 platelets during 12 min (Figure 5A). Figure 5B shows the multiple comparison with F/F_0_ distribution plot of 20 platelets in each group at the time of 8.5 min. F value from ANOVA analysis among these five groups is 19.547, *p* < 0.001. Exposure of platelets to shear at a rate of 1,000 s^−1^ induced a robust Ca^2+^ increase in platelets, and such Ca^2+^ response was enhanced by treatment with Yoda1 (*p* = 0.03) and, conversely, was strongly suppressed by treatment with GsMTx-4 (*p* < 0.01) These results are highly consistent with the notion that shear-induced activation of the Piezo1 channel results in an increase in the Ca^2+^ concentration in platelets as reported by a recent study (Ilkan et al., 2017). Similarly, shear-induced Ca^2+^ response was strongly suppressed by treatment with XST (0.15 g L^−1^). Such treatment with XST also significantly reduced Ca^2+^ responses induced by shear and Yoda1. These results provide evidence to show that XST inhibits shear-induced activation of the Piezo1 channel and thereby shear-induced Ca^2+^ signaling in platelets.

### Effects of XueShuanTong on Platelet Aggregation Related Proteins Expressions Under High-Shear Stress

**FIGURE 6:**
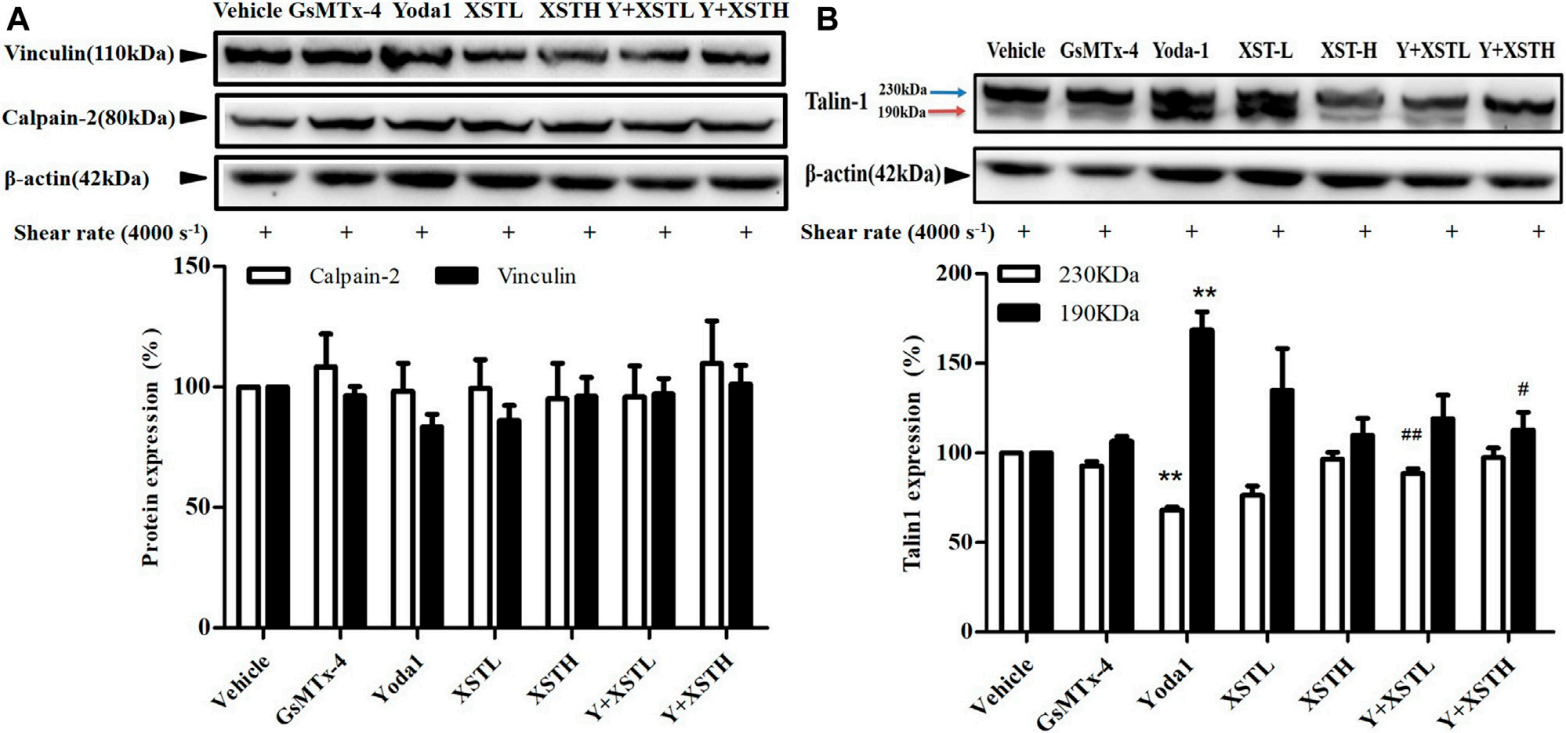
Effects of XST on the expression of platelet aggregation-related proteins under shear stress. **(A)** Western blotting analysis of the expression of calpain-2 and vinculin in platelets subjected to shear at a rate of 4,000 s^−1^ and treated with saline, GsMTx-4 (2.5 µM), Yoda1 (25 µM) and XST at 0.15 g L^−1^ (XSTL) or 0.6 g L^−1^ (XSTH). Treatment with XST resulted in no significant alteration in the expression of calpain-2 and vinculin. **(B)** Western blotting analysis of talin1 cleavage in platelets subjected to shear at a rate of 4,000 s^−1^ and treated with saline, GsMTx-4 (2.5 µM), Yoda1 (25 µM), and Yoda1 together with XST at 0.15 g L^−1^ or 0.6 g L^−1^ highlighted by black lines. Top: representative blots; bottom: mean data from three independent experiments. *p < 0.05, **p < 0.01 compared to platelets subjected to shear alone; ^#^ *p* < 0.05, ^##^ *p* < 0.01 compared to platelets treated with Yoda1 alone highlighted by black lines.

**FIGURE 7:**
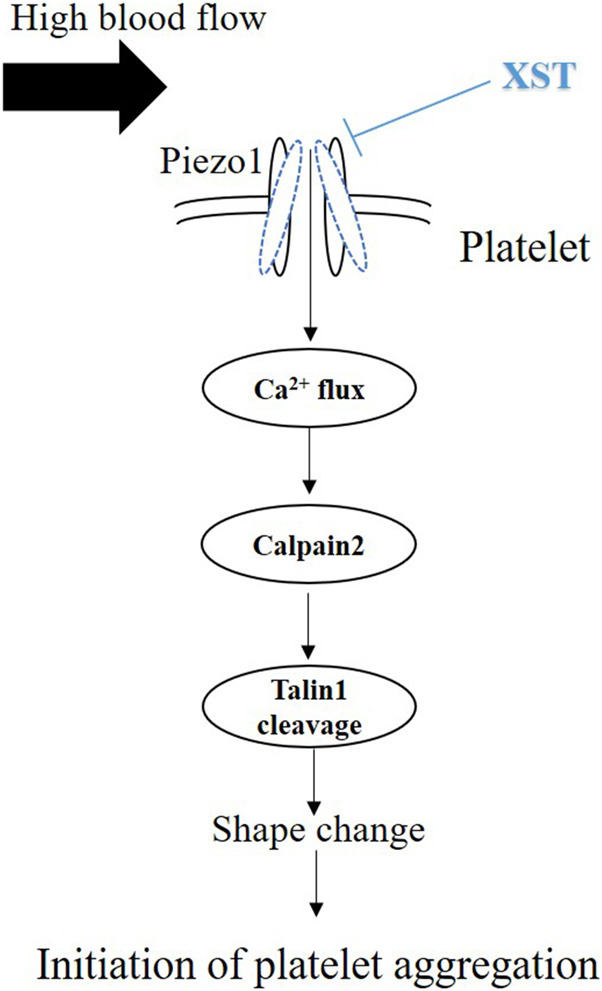
Schematic summary of inhibition by XST of shear-induced platelet aggregation. Exposure to shear activates the Piezo1 channel and ensuing Piezo1-mediated Ca^2+^ influx. An increase in intracellular Ca^2+^ level in turn stimulates calpain-2 and cleavage of talin1, leading to platelet aggregation. Exposure to high shear rate also upregulates the expression of Pizo1 channel (not depicted here). Treatment with XST at a clinically relevant concentration inhibits shear-induced platelet aggregation via targeting the Piezo1 channel.

A recent study has shown calpain activation downstream of shear-induced Piezo1-mediated Ca^2+^ signaling in endothelial cells (Li et al., 2014). Several Ca^2+^ responsive proteins have been identified that control this inside-out integrin activation. Activation of Ca^2+^ dependent protease calpain and cleavage of its target talin was demonstrated after Piezo1 activation in epithelial cells (McHugh et al., 2010). Calpain catalyzing activity was significantly increased upon Yoda1 treatment (Aglialoro et al., 2020) and calpain cleaves talin to an activated state mediating inside-out integrin activation (Goksoy et al., 2008; McHugh et al., 2010). We therefore performed western blotting to examine whether treatment with XST altered the expression of calpain-2 and vinculin, the downstream signals of Piezo1, which are known to be involved in platelet activation and aggregation (Serrano and Devine, 2004; Kuchay and Chishti, 2007). Neither calpain-2 nor vinculin protein level in platelets was altered by treatment with shear, Yoda1 or GsMTx-4. Treatment with XST resulted in no significant alteration in the expression of calpain-2 and vinculin (Figure 6A). Calpain-2 catalyzes cleavage talin1 protein from its 230 kDa isoform to a smaller 190 kDa isoform, which is recruited to form a complex with the GPIII_b_III_a_ protein to induce activation of platelets. Western blotting analysis indicated that cleavage of talin1 in platelets under shear with a rat of 4,000 s^−1^ was inhibited by treatment with MDL28170, a calpain-2 inhibitor (Supplementary Figure S6), suggesting shear-induced activation of calpain-2 in platelets. In platelets under such shear condition, treatment with Yoda1 enhanced a reduction of the 230 kDa protein level and a simultaneous increase in the 190 kDa protein level, while treatment with GsMTx-4 had no effect (Figure 6B). These results implied that synergic stimulation of the Piezo1 channel activity by shear and Yoda1 led to activation of Calpain-2 and subsequent cleavage of talin1 (Figure 6B). Treatment together with XST significantly attenuated the reduction in the 230 kDa protein level or the increase in the 190 kDa protein level, suggesting XST inhibits shear/Yoda1-induced activation of calpain-2 and subsequent cleavage of talin1 (Figure 6B). These results are consistent with that XST inhibits shear-induced platelet aggregation via Piezo1-mediated Ca^2+^ signaling and subsequent calpain-2 activation and talin1 cleavage as downstream signal pathways (Figure 7).

## Discussion

The aim of this study is to explore the effects and mechanism of XST on shear-induced platelet aggregation. Microfluidic system was used to generate controlled shear stress on platelet aggregation. Numerous recent studies have revealed an important role of the Ca^2+^-permeable Piezo1 channel in sensing mechanical forces and regulating a bewildering number of physiological processes including vascular development, vascular tone and blood flow (e.g., Li et al., 2014; Wang et al., 2016; Beech, 2018). Mechanical activation of the Piezo1 channel is essential for homeostasis of red blood cell (Cahalan et al., 2015). It has been shown in a recent study that Piezo1 functions as a mechanosensitive channel in human platelets that mediates arterial shear-induced Ca^2+^ entry and thrombus formation (Ilkan et al., 2017). Shear stress activates Piezo1 (Douguet et al., 2019), and the opening of endothelial Piezo1 channels by shear stress activates downstream Ca^2+^-dependent calpains, which in turn are responsible for focal adhesion turnover, proteolytic cleavage of actin cytoskeletal elements, and alignment of ECs to blood flow (Beech, 2018). In this study, we showed an increase in the Ca^2+^ level in platelets in response to a wide range of physiologically and pathologically relevant shear (Figure 5). Such Ca^2+^ responses were inhibited by treatment with GsMTx-4 to inhibit the Piezo1 channel or further enhanced by treatment with Yoda1 (Figure 5). Interestingly, western blotting analysis also revealed that exposure to high shear significantly unregulated the expression of the Piezo1 channel (Figure 3C). Such shear-induced regulation of the Piezo1 channel expression was insensitive to treatment with GsMTx-4, suggesting engagement of a Piezo1-independent mechanism which requires further investigation. Overall, our results indicate that shear induces activation of the Piezo1 channel leading to Ca^2+^ influx in platelets. In the present study we also showed that exposure to physiological and pathological shear stimulated platelet aggregation that was strongly suppressed by treatment with GsMTx-4 (Figure 2), an inhibitor of Piezo1 channel, providing evidence to suggest that shear-induced platelet aggregation via activating the Piezo1 channel.

Piezo1 channels may directly function as shear stress sensors involved in perceiving shear stress and critical for calcium influx and subsequent integrin activation. In endothelial cells, Piezo1 activation has been associated with integrin activation and increased cell adhesion resulting in integrin-dependent focal adhesion kinase signaling under flow shear stress. Reduced integrin activation was observed in an endothelium-specific Piezo1 deficient mouse model (Albarrán-Juárez et al., 2018). In agreement with this, depletion of Piezo1 in small lung cancer cell lines also caused decreased integrin activation (McHugh et al., 2012). Activation of Piezo1 on erythroblasts by Yoda1 causes activation of downstream Ca^2+^ mediators calpain and PKC, resulting in inside-out activation of integrins (Aglialoro et al., 2020). Also several Ca^2+^ responsive proteins have been identified that control this inside-out integrin activation, for instance, the Ca^2+^ dependent calpain unmasks the β-integrin binding motif of talins leading to integrin activation (Goksoy et al., 2008; McHugh et al., 2010). It is well known that the Ca^2+^ signaling plays an important role in platelet activation and aggregation. Studies have reported that shear-induced Piezo1-mediated Ca^2+^ signal activates calpain-2 (Miyazaki et al., 2010; Li et al., 2014). Calpain-2 is a calcium-dependent protease, meanwhile calcium influx activates calpain-2. Talin-1, one of calpain-2 substrates, is a large (∼2,540 residues) dimeric adaptor protein that associates with the integrin family of cell adhesion molecules in cell-extracellular matrix junctions, where it both activates integrin and couples them to the actin cytoskeleton. Calpain cleaves talin to an activated state mediating inside-out integrin activation (Goksoy et al., 2008; McHugh et al., 2010). Calpain-2 has been implicated in the modulation of integrin signaling, particularly the αIIbβ_3_ (GPIIb/IIIa) integrin-mediated signaling during platelet activation. Calpain-2 regulates adhesion turnover and disassembly of platelets through targeting specific substrates, such as talin1 (Franco et al., 2004) and vinculin (Serrano and Devine, 2004), thereby affecting morphology of platelets. There is evidence to suggest binding of talin1 to the β_3_ integrin results in activation of integrin αIIbβ3 and while activation of αIIbβ3 also depends on the activity of calpain to cleave talin1 (Petrich et al., 2007; Watanabe et al., 2008). Calpain-2 cleaves 230 kDa talin1 into 190 kDa. Calpain activity was significantly increased upon Yoda1 treatment, and Yoda1 led to a modest but clear increase in cleaved activated talin (Aglialoro et al., 2020), which is agreement with our finding that Yoda1 treatment led to a significant increase in talin cleavage (Figure 6B).

We showed that exposure to shear resulted in no effect on the protein expression of calpain-2 and vinculin in platelets (Figure 6A). However, treatment with calpain-2 inhibitor MDL28170 under shear rate of 4,000 s^−1^ significantly suppressed cleavage of talin1 (Supplementary Figure S6), suggesting that shear stimulates calpain-2 activity and then cleavage of talin1. Yoda1 showed no direct effects on calpain-2 protein expression level, however, platelets treated with Yoda1 under shear rate of 4,000 s^−1^, enhanced talin1 cleavage and increased its 190 kDa protein isoform, which is recruited to form a complex with the GPIIbIIIa protein to induce activation of platelets, with implications for thrombosis. These results imply that shear-induced platelets aggregation is through piezo1 channel-mediated calcium influx and calcium-dependent activation of calpain-2 and subsequent talin1 cleavage. Treatment with XST significantly attenuated the reduction in the 230 kDa protein level or the increase in the 190 kDa protein level (Figure 6B), suggesting XST inhibits shear induced activation of calpain-2 and subsequent cleavage of talin1. Taken together, our results suggest that XST inhibits shear-induced platelet aggregation via Piezo1-mediated Ca^2+^ signaling and subsequent calpain-2 activation and talin1 cleavage as downstream signal pathways (Figure 7).

The results from this study implies that XST with clinical concentrations might inhibit shear-induced platelet aggregation via Piezo1 channel and calcium influx. As introduced above, XST has been approved for its use in TCM to treat thrombosis. The recommendation of clinical dosage of XST is 500 mg via intravenous administration per day, which is approximately equal to 0.10–0.15 g L^−1^ plasma concentration. According to the human pharmacokinetics of XST study, these ginsenosides exhibited increased maximum plasma total concentration (C_max_) and area under the plasma concentration–time curve from 0 to infinity (AUC_0–∞_) as the XST dose increased from 250 to 500 mg/person. Intravenous repeatedly dosing of 500 mg XST for 15 days, the C_max_ of major ginsenosides is approximately 0.09–0.10 g L^−1^ (Pintusophon et al., 2019). In our previous study, 0.15 g L^−1^of XST showed significant effects on platelets activation and aggregation (Han et al., 2019). In this study, we showed that treatment with XST at 0.15 g L^−1^ remarkably inhibited shear-induced platelet aggregation and treatment with XST at a high concentration of 0.6 g L^−1^ resulted in slightly greater inhibition (Figure 3). XST at 0.15 g L^−1^ also potently suppressed platelet aggregation induced by elevated activation of the Piezo1 channel using Yoda1 in the presence of shear stress (Figure 4). However, treatment with XST even at the high concentration used in this study did not change high shear induced vWF-mediated platelet adhesion and rolling (Supplementary Figure S5). Taken together, these results indicate that XST at a clinically relevant concentration preferentially inhibits shear-induced platelet aggregation without effect on interactions between platelet and vWF. We further demonstrated using single Ca^2+^ imaging that treatment with XST at 0.15 g L^−1^ was effective in inhibiting Piezo1-mediated Ca^2+^ response in platelets induced by shear or combination of shear and Yoda1, like treatment with GsMTx-4 (Figure 5). Consistently, we showed using western blotting analysis that treatment with XST significantly Yoda1-induced activation of calpain-2 and cleavage of talin1 (Figure 6B). Collectively, our results suggest that XST at a clinically relevant concentration inhibits shear-induced platelet aggregation via Piezo1-mediated Ca^2+^ signaling and downstream signal pathway (Figure 7), providing novel insights into the molecular mechanisms underpinning the therapeutic effects of XST.

In summary, our study shows that high shear induced platelet aggregation might be via Piezo1-mediated Ca^2+^ signaling and downstream signal pathway and XST at clinically relevant concentrations inhibit shear-induced platelet aggregation by preventing shear-induced activation of the Piezo1 channel.

## Data Availability

The original contributions presented in the study are included in the article/Supplementary Material, further inquiries can be directed to the corresponding authors.
