# Supplementary material for: Inhibition of Shear-Induced Platelet Aggregation by Xueshuantong via Targeting Piezo1 Channel-Mediated Ca2+ Signaling Pathway
Source: Front Pharmacol. 2021 Mar 22;12:606245. doi: 10.3389/fphar.2021.606245 (PMC8025832; doi:10.3389/fphar.2021.606245)
Supplement: Supplementary file 1 [file Image1.jpeg]

Frontiers | Inhibition of shear-induced platelet aggregation by Xueshuantong via targeting Piezo1 channel-mediated Ca 2+ signalling pathway | Pharmacology


- About
- Journals
- Research Topics
- Articles
- More

Submit

My Frontiers

Office

- TSOF
  - TSOF
  - Article Production

Typesetter 3

frontiersproduction@tnq.co.in

- Profile
- Settings & Privacy
- Help Center
- Logout

Submit

**Impact Factor 4.225** | **CiteScore 5.0**More on impact ›

|  |  |
| --- | --- |
| Frontiers in Pharmacology | Cardiovascular and Smooth Muscle Pharmacology |

Toggle navigation


Section


- (current)Section
- About
- Articles
- Research topics
- For authors 
  - Why submit?
  - Fees
  - Article types
  - Author guidelines
  - Review guidelines
  - Submission checklist
  - Contact editorial office
  - Submit your manuscript
- Editorial board

- *Article alerts*

Articles


**Suggest a Research Topic >**

- 57
  total views

 View Article Impact

**Suggest a Research Topic >**

##### SHARE ON

- Facebook

  0
- Twitter

  0
- LinkedIn

  0
- AddThis

  New


## Original Research ARTICLE

Front. Pharmacol.
| doi: 10.3389/fphar.2021.606245

# Inhibition of shear-induced platelet aggregation by Xueshuantong via targeting Piezo1 channel-mediated Ca 2+ signalling pathway Provisionally accepted The final, formatted version of the article will be published soon. **Notify me**

Lei Liu1, Qiongling Zhang1, 
Ying Chen1, 
 Ding S. Lan1,  Zhengxiao Sun1, Lan Wang1, 
Xiaojie Yin1, 
Fulong Liao1, 
 Lin-Hua Jiang2, Mei Xue3\* and 
 You YUN1\*

- 1Institute of Chinese Materia Medica, China Academy of Chinese Medical Sciences, China
- 2Multidisciplinary Cardiovascular Research Centre, Faculty of Medicine and Health, University of Leeds, United Kingdom
- 3Xiyuan Hospital, China Academy of Chinese Medical Sciences, China

XueShuanTong (XST) comprising therapeutically active ginsenosides, a lyophilized extract of Panax notoginseng roots, is extensively used in traditional Chinese medicine to treat ischemic heart and cerebrovascular diseases. Our recent study shows that treatment with XST inhibits shear-induced thrombosis formation but the underlying mechanism remained unclear. This study aimed to investigate the hypothesis that XST inhibited shear-induced platelet aggregation via targeting the mechanosensitive Ca2+-permeable Piezo1 channel by performing platelet aggregation assay, Ca2+ imaging and Western blotting analysis. Exposure to shear at physiologically (1000-2000 s-1) and pathologically related rates (4000-6000 s-1) induced platelet aggregation that was inhibited by treatment with GsMTx-4. Exposure to shear evoked robust Ca2+ responses in platelets that were inhibited by treatment with GsMTx-4 and conversely enhanced by treatment with Yoda1. Treatment with XST at a clinical relevant concentration (0.15 g·L-1) potently inhibited shear-induced Ca2+ responses and platelet aggregation, without altering vWF-mediated platelet adhesion and rolling. Exposure to shear, while resulting in no effect on the calpain-2 expression in platelets, induced calpain-2-mediated cleavage of talin1 protein, which is known to be critical for platelet activation. Shear-induced activation of calpain-2 and cleavage of talin1 were attenuated by treatment with XST. Taken together, our results suggest that XST inhibits shear-induced platelet aggregation via targeting the Piezo1 channel to prevent Piezo1-mediated Ca2+ signalling and downstream calpain-2 and talin1 signal pathway, thus providing novel insights into the mechanism of the therapeutic action of XST on platelet aggregation and thrombosis formation.

Keywords: 
Xueshuantong, shear, Platelet Aggregation, Piezo1 channel, Ca2+

Received: 13 Oct 2020;
Accepted: 08 Feb 2021.

Copyright: © 2021 Liu, Zhang, Chen, Lan, Sun, Wang, Yin, Liao, Jiang, Xue and YUN. This is an open-access article distributed under the terms of the Creative Commons Attribution License (CC BY). The use, distribution or reproduction in other forums is permitted, provided the original author(s) and the copyright owner(s) are credited and that the original publication in this journal is cited, in accordance with accepted academic practice. No use, distribution or reproduction is permitted which does not comply with these terms.

\* Correspondence: 
  
 Prof. Mei Xue, Xiyuan Hospital, China Academy of Chinese Medical Sciences, Beijing, 100091, China, meiar@126.com   
 Prof. You YUN, Institute of Chinese Materia Medica, China Academy of Chinese Medical Sciences, Beijing, China, youyunrice@126.com

Write a comment...

Add

##### COMMENTARY

##### ORIGINAL ARTICLE

##### People also looked at

## Long non-coding RNA TRPM2-AS promotes the growth, migration and invasion of retinoblastoma via miR-497/WEE1 axis

Aipeng Li, Jingpu Yang, Ting Zhang, Lin Li and Miyang Li

## ICU patients' antibiotic exposure and triazole-resistance in invasive candidiasis: parallel analysis of aggregated and individual data

Yan Wang, Ying Zhang, Treasure M Mcguire, Samantha A Hollingworth, Mieke L Van Driel, Lu Cao, Xue Wang and Yalin Dong

## Predisposition to Alzheimer’s and Age-Related Brain Pathologies by PM2.5 Exposure: Perspective on the Roles of Oxidative Stress and TRPM2 Channel

Lu Wang, Lin Yu Wei, Ran Ding, Yanyan Feng, Dongliang Li, Chaokun Li, Philippa Malko, Sharifah A. Syed Mortadza, Weidong Wu, Yaling Yin and Lin-Hua Jiang

**Suggest a Research Topic >**

×

#### Supplementary Material

  

There is no supplementary material currently available for this article

Loading supplemental data...

  

|  | File Name |  |
| --- | --- | --- |
|  | Video 1.MP4 |  |
|  | Video 2.MP4 |  |
|  | Video 3.MP4 |  |
|  | Video 4.MP4 |  |
|  | Video 5.MP4 |  |
|  | Video 6.MP4 |  |
|  | Video 7.MP4 |  |
|  | Data Sheet 2.docx |  |
|  | Data Sheet 3.DOCX |  |
|  | Data Sheet 4.ZIP |  |
|  | Data Sheet 5.ZIP |  |
|  | Data Sheet 6.ZIP |  |
|  | Data Sheet 7.ZIP |  |
|  | Data Sheet 8.ZIP |  |
|  | Image 1.JPEG |  |
|  | Image 2.JPEG |  |
|  | Image 3.JPEG |  |
|  | Image 4.JPEG |  |
|  | Image 5.JPEG |  |
|  | Image 6.JPEG |  |

  

Close

- About Frontiers
- Institutional Membership
- Books
- News
- Frontiers' social media
- Contact
- Careers
- Submit
- Newsletter
- Help Center
- Terms & Conditions
- Privacy Policy

© 2007 - 2021 Frontiers Media S.A. All Rights Reserved

### Privacy Preference Center

Our website uses cookies that are necessary for its operation. Additional cookies are only used with your consent. These cookies are used to store and access information such as the characteristics of your device as well as certain personal data (IP address, navigation usage, geolocation data) and we process them to analyse the traffic on our website in order to provide you a better user experience, evaluate the efficiency of our communications and to personalise content to your interests. Some cookies are placed by third-party companies with which we work to deliver relevant ads on social media and the internet. Click on the different categories' headings to change your cookie preferences. Click on "More Information" if you wish to learn more about how data is collected and shared.
More information

### Manage Consent Preferences

#### Strictly Necessary Cookies

Always Active

These cookies are necessary for the website to function and cannot be switched off in our systems. They are usually only set in response to actions made by you which amount to a request for services, such as setting your privacy preferences, logging in or filling in forms. You can set your browser to block or alert you about these cookies, but some parts of the site will not then work. These cookies do not store any personally identifiable information.

#### Analytics Cookies

Analytics Cookies

These cookies allow us to count visits and traffic sources so we can measure and improve the performance of our site. They help us analyse which pages are the most and least popular and see how visitors move around the site.    All information these cookies collect is aggregated and therefore anonymous.

#### Functional Cookies

Functional Cookies

These cookies enable the website to provide enhanced functionality and personalisation. They may be set by us or by third party providers whose services we have added to our pages. If you do not allow these cookies then some or all of these services may not function properly.

#### Advertising Cookies

Advertising Cookies

These cookies may be set through our site by our advertising partners. They may be used by those companies to build a profile of your interests and show you relevant adverts on other sites.    They do not store directly personal information, but are based on uniquely identifying your browser and internet device. If you do not allow these cookies, you will experience less targeted advertising.

### Back Button Performance Cookies

Vendor Search  Search Icon

Filter Icon

Clear

checkbox label label

Apply Cancel

Consent Leg.Interest

checkbox label label

checkbox label label

checkbox label label

Confirm My Choices
