# Supplementary material for: Inhibition of Shear-Induced Platelet Aggregation by Xueshuantong via Targeting Piezo1 Channel-Mediated Ca2+ Signaling Pathway
Source: Front Pharmacol. 2021 Mar 22;12:606245. doi: 10.3389/fphar.2021.606245 (PMC8025832; doi:10.3389/fphar.2021.606245)
Supplement: Supplementary file 6 [file datasheet2.docx]

Supplementary Material

**S Figure 1** **The Bioflux1000Z Microfluidic Culture System.**

(**A**) Bioflux1000Z Instrument components. From left to right are the automated Zeiss microscope with the CCD camera, PC Workstation, BioFlux Controller and Fluorescence Module. (**B**) BioFlux Plate. This plate features 24 experimental channels, each with an input and output well. (**C**) Principles of Operation. The flow channels can be coated with proteins, adhesion molecules and cellular monolayers to conduct a wide range of cell-cell and cell-ligand interaction assays. (**D**) Viewing Window.

**S Figure 2** **BioFlux 24-well Plate**

This plate features 8 experimental channels, each with two input wells and one outlet well.

**S Figure 3** **Effects of XST on vWF-mediated platelet adhesion and rolling under shear**

Mean adhesion **(A)** and rolling velocity **(B)** of platelets under control conditions or treatment with XST at 0.15 g·L^-1^(XSTL) or 0.6 g·L^-1^ (XSTH).

**S Figure 4 Effect of treatment with** **MDL28170 on cleavage of talin1 in platelets under shear**

Western blotting analysis of talin1 cleavage in platelets subjected to shear at a rate of 4000 s^-1^ and treated with saline or MDL28170 (250 µM). Top: representative blots; bottom: mean data from 3 independent experiments. ***P*<0.01 compared to platelets subjected to shear alone.

**S Figure 5 Effect of treatment with Aspirin on shear induced platelet aggregation and Piezo1 expression under shear stress**

**(A)** and **(B)** Western blotting analysis of Piezo1 expression in platelets subjected to shear at a rate of 1000 s^-1^ and 10000 s^-1^ treated with saline or Aspirin (2.5 µM). Top: representative blots; bottom: mean data from 3 independent experiments. **P*<0.05 compared to platelets without subjected to shear.

**(C)** Platelets aggregation rate detected by Bioflux 1000z microfluidic system under shear rate of 1000 and 8000 s^-1^ for five minutes respectively. There were no significant differences between vehicle and Aspirin (2.5 µM) treatment on shear induced platelet aggregation. Mean data from 3 independent experiments.

**S Figure 6 Chemical analysis data of XST with Lot No. 17081207 detected and provided by Guangxi Wuzhou Pharmaceutical Group**

**S Figure 7 Effects of DMSO on shear induced platelets aggregation**

(A) Representative images showing effects of treatment with Saline (0.25% v/v), DMSO (0.25% v/v) and DMSO (1.0% v/v) on platelet aggregation under shear rates of 400, 1000, 2000, 4000 and 8000 s^-1^. The scale bar is 50μm. (B) Mean platelet aggregation rate at the end of exposure to indicated shear

rates from three independent experiments, **P*<0.05, ***P*<0.01 and “ns” means no significant, analyzed by two-way ANOVA and post-hoc Tukey’s test.

**Video** **Platelet aggregation under varying shear rates**

PRP loaded with calcein-AM were perfused over collagen-coated surface. See Figure 1 legend to for details. The movie shows platelet aggregation under shear with the rate increased from 400 s^-1^ to 8000 s^-1^. Images obtained with an Axio objective7 (Zeiss) and digital camera (Hamamatsu C11440). Treatments: (a) vehicle; (b) GsMTx-4; (c) XST-L; (d) XST-H; (e) Yoda1; (f) Yoda1 together with XST-L; (g) Yoda1 together with XST-H.
